# Relationship of Adipocyte Size with Adiposity and Metabolic Risk Factors in Asian Indians

**DOI:** 10.1371/journal.pone.0108421

**Published:** 2014-09-24

**Authors:** Ved Prakash Meena, V. Seenu, M. C. Sharma, Saumya Ranjan Mallick, Ashu Seith Bhalla, Nandita Gupta, Anant Mohan, Randeep Guleria, Ravindra M. Pandey, Kalpana Luthra, Naval K. Vikram

**Affiliations:** 1 Department of Medicine, All India Institute of Medical Sciences, New Delhi, India; 2 Department of Surgical Disciplines, All India Institute of Medical Sciences, New Delhi, India; 3 Department of Pathology, All India Institute of Medical Sciences, New Delhi, India; 4 Department of Radiodiagnosis, All India Institute of Medical Sciences, New Delhi, India; 5 Department of Endocrinology and Metabolism, All India Institute of Medical Sciences, New Delhi, India; 6 Departments of Pulmonary Medicine and Sleep Disorders, All India Institute of Medical Sciences, New Delhi, India; 7 Department of Biostatistics, All India Institute of Medical Sciences, New Delhi, India; 8 Department of Biochemistry, All India Institute of Medical Sciences, New Delhi, India; Complexo Hospitalario Universitario de Santiago, Spain

## Abstract

**Background:**

Enlargement of adipocyte is associated with their dysfunction and alterations in metabolic functions.

**Objectives:**

We evaluated the association of adipocyte size of subcutaneous and omental adipose tissue with body composition and cardiovascular risk factors in Asian Indians.

**Methodology:**

Eighty (40 males and 40 females) non-diabetic adult subjects undergoing elective abdominal surgery were included. Pre-surgery evaluation included anthropometric measurements, % body fat by bioimpedance, abdominal fat area at L_2–3_ level (computed tomography) and biochemical investigations (fasting blood glucose and insulin, lipids and hsCRP). During surgery, about 5 grams each of omental and subcutaneous adipose tissue was obtained for adipocyte size determination.

**Results:**

Females had higher BMI, % body fat, skinfold thickness, total and subcutaneous abdominal fat area as compared to males. Overweight was present in 42.5% and 67.5%, and abdominal obesity in 5% and 52.5% males and females, respectively. Subcutaneous adipocyte size was significantly higher than omental adipocyte size. Omental adipocyte size correlated more strongly than subcutaneous adipocyte size with measures of adiposity (BMI, waist circumference, %BF), total and subcutaneous abdominal fat area and biochemical measures (fasting glucose, total cholesterol, triglycerides and HOMA-IR), the correlations being stronger in females. The correlation of adipocyte size with metabolic parameters was attenuated after adjusting for measures of adiposity.

**Conclusion:**

Omental adipocyte size, though smaller than the subcutaneous adipocyte size, was more closely related to measures of adiposity and metabolic parameters. However, the relationship was not independent of measures of adiposity.

## Introduction

Obesity is a medical condition with accumulation of excess body fat adversely affecting health leading to reduced life expectancy and/or increased health problems. It may be defined as an abnormal growth of the adipose tissue due to an enlargement of fat cell size (hypertrophic obesity) or an increase in fat cell number (hyperplasic obesity) or a combination of both [1]. The prevalence of obesity, particularly abdominal obesity, is increasing rapidly all over the world, including developing countries [2]. Obesity increases the risk of various diseases, including cardiovascular disease, type 2 diabetes, sleep disordered breathing, certain types of cancer, and osteoarthritis [3]. Abdominal obesity is more closely related to insulin resistance, metabolic syndrome and type 2 diabetes [4].

Asian Indians have been shown to be more insulin resistant at any given body mass index (BMI) and are more likely to develop type 2 diabetes and cardiovascular disease as compared to their European counterparts [5], [6]. Some unique phenotypic features among Asian Indians, such as increased body fat, abdominal and truncal adiposity may contribute to this increased insulin resistance [4], [7]. These features appear to be present from birth as Indian neonates tend to have increased body fat compared to their white Caucasian counterparts despite lower birth weights [8]. These features have been referred to as the “Asian Indian Phenotype or Paradox”.

Not all obese subjects develop metabolic abnormalities. Some observations indicate that being moderately overweight may actually be protective against mortality due to non-cancerous and non-cardiovascular cause and have lower mortality from cancer and cardiovascular disease (CVD) [9]. However, a recent systematic review indicates that metabolically healthy overweight and obese individuals may be at increased risk for adverse long-term outcomes [10]. The risk of development of abnormalities may be determined by the number and size of adipocytes (hyperplastic vs. hypertrophic obesity). In the presence of positive calorie balance, the mature adipocytes initially undergo enlargement, which acts as a trigger for recruitment and proliferation of preadipocytes to add more functional adipocytes [11], [12], [13]. If the process of adipogenesis is impaired after initial hypertrophy, it leads to adipose tissue metabolic and immunologic dysfunction [14], [15].

Adipose tissue is not a mere depot for fat storage but is an active endocrine organ and secretes various adipocytokines which include adiponectin, leptin, resistin, and proinflammatory cytokines like tumor necrosis factor alpha (TNF-α) which modulate insulin sensitivity and related metabolic abnormalities. Enlargement of adipocyte is associated with substantial changes in metabolic functions [16]. Several reports have indicated that adipocyte size of the visceral and subcutaneous adipose tissue is associated with insulin resistance. In their study, Weyer *et al.* observed that large subcutaneous adipocytes predicted insulin resistance and type 2 diabetes, independent of obesity in those with normal glucose tolerance (NGT) [17]. Furthermore, In another study involving women, subcutaneous adipocyte size, particularly in the abdominal region, in addition to the amount and distribution of body fat, predicted incidence of type 2 diabetes in later life [18].

Majority of these studies have been conducted on populations other than Asian Indians and a few studies have included migrant Asian Indians. A study involving migrant Asian Indians indicated that subcutaneous fat is a better determinant of insulin resistance than visceral fat [19]. There is paucity of data on Asian Indians from India in this regard. Therefore the present study aimed to evaluate the association of adipocyte size of subcutaneous and omental adipose tissue with body composition, insulin resistance and subclinical inflammation in non-diabetic adult Asian Indian subjects.

## Materials and Methods

### Study population

This cross-sectional study was undertaken in the Department of Medicine, All India Institute of Medical Sciences, New Delhi, India from 2010 to 2012. The study was approved by the Institutional Ethics Committee of All India Institute of Medical Sciences, New Delhi, and written informed consent was obtained from subjects prior to inclusion in the study. Eighty adult subjects (40 males and 40 females) in the age group of 20–60 years, undergoing elective abdominal surgery in the General Surgery Department of the institute were included in the study. Exclusion criteria included any known malignancy, known seropositivity for HIV, HBV or HCV, any severe acute or chronic medical or surgical illness, pregnancy and lactation.

### Measurements

#### Anthropometry and Biochemical parameters

Subjects underwent detailed clinical evaluation before surgery including detailed clinical history, physical examination and anthropometric measurements (height, weight, body mass index [BMI], waist (WC) and hip (HC) circumferences, waist to hip circumference ratio (WHR) mid-arm and mid-thigh circumferences and skinfold thickness measurements) according to the methods mentioned elsewhere [20]. A venous blood sample was obtained after an overnight fast of at least 10 hours. Biochemical measurements included fasting blood glucose (FBG), total cholesterol (TC), triglycerides (TG), high density lipoprotein cholesterol (HDL-c) and hsCRP levels (using ELISA) performed according to the methods described earlier [20]. Value of low density lipoprotein cholesterol (LDL-c) was calculated using Freidewald's equation [21]. Fasting insulin levels were measured using an eletrochemieluminometric assay on a Roche ELECSYS e411 auto analyser.

#### Percentage body fat and abdominal fat

Percentage body fat was measured by bioelectrical impedance method (TANITA CORP, JAPAN). Abdominal fat area (cm^2^) was determined by computed tomography scan at the level of L_2–3_. Total (TAF), subcutaneous (SAF) and visceral (VAF) abdominal fat area was measured in L_2–3_ slice by mapping various adipose tissue compartments on the computer screen using a track-ball.

#### Adipocyte size measurement

In patients who underwent cholecystectomy, subcutaneous (SC) adipose tissue was obtained through a 1 cm transverse incision given about 2 cm below the xiphisternum in the epigastric region to the right of midline. In patients undergoing incisional hernia repair, subcutaneous adipose tissue was obtained through a 1 cm transverse incision given about 2 cm below the umbilicus to the right of midline. Omental (OM) adipose tissue was obtained from the free edge of the greater omentum in all the patients. At the time of surgery, about 5 grams of each OM and SC adipose tissue was obtained and was fixed in 10% neutral buffered formalin. Subsequently, it was routinely processed and 5 µm thick sections were cut and stained with haematoxylin and eosin. Microphotographs were captured at 200 magnification using DP 70 digital camera with Olympus BX51 microscope (M/S –Japan). The images were processed with image pro plus software (image proplus version 7, mediacybernetics USA). Fifty non-overlaping adipocytes were traced in semiautomatic manner. Area and maximum diameter were calculated and the data was transported to Microsoft excel. In about 20% of samples there were less than 100 non-overlapping adipocytes for morphometric analysis, therefore for uniformity, 50 non-overlapping adipocytes were taken for measurements. Furthermore, comparison of adipocyte measurements (mean area and diameter) obtained by analyzing 100 adipocytes versus 50 adipocytes in the same individual did not reveal any significant difference.

### Definitions

Overweight was defined as BMI≥23 kg/m^2^ [22]. Abdominal obesity was defined as WC>90 cm in males and>80 cm in females [23]. Dyslipidemias were defined as per the criteria of National Cholesterol Education Program, Adult Treatment Panel III (NCEP, ATP III) [24]. The metabolic syndrome was defined as the presence of at least three of the following abnormalities: FBG≥100 mg/dL, central obesity (as defined by WC>90 cm in males and>80 cm in females), hypertriglyceridemia (TG≥150 mg/dL), low HDL-c (<40 mg/dL in males and <50 mg/dL in females) and blood pressure ≥130/85 mmHg [25]. Impaired fasting glucose (IFG) was defined as FBG 100 to125 mg/dL. High levels of hs-CRP were defined as>3 mg/L [26]. Hyperinsulinemia was defined as levels>10.4 µU/mL. Insulin resistance was determined by the homeostatic model assessment (HOMA-IR) method (fasting glucose [mmol]×fasting insulin [µU/mL]/22.5) [27]. A value of HOMA-IR>2.29 was defined as high indicating insulin resistance [28].

### Statistical analysis

Comparison of normally distributed variables among male and female subjects was done by student's ‘t’ test. Comparison of non-normally distributed variables was done by using Wilcoxon ranksum test. Correlations of omental and subcutaneous adipocyte size with body composition and metabolic parameters were evaluated by Spearman's Ranksum correlation coefficient. Multivariate regression analysis was performed to assess whether adipocyte size is correlated with metabolic parameters independent of body composition. Statistical analysis was performed using STATA software (version 9; StataCorp, College Station, TX) and a P value<0.05 was taken as statistically significant.

## Results

A total of 132 patients were screened during the time period of June 2010 to May 2012, out of which 80 patients fulfilled the criteria of the study and were included. Out of these, 73 patients were having gall stone disease and 7 patients were having incisional hernia. Fifty two patients were excluded due to malignancy, diabetes mellitus, existing coronary artery disease, liver or kidney disease and 13 patients didn't give consent.

### Clinical, anthropometric, body fat and biochemical characteristics (Table 1)

Females were older, had higher BMI, HC and skinfold thickness than males. Mean values of systolic and diastolic blood pressures were comparable among males and females. Mean values of FBG, TC and HDL-c were significantly higher in females as compared to males, whereas serum levels of TG were significantly higher in males. Values of fasting insulin, hsCRP and HOMA-IR were comparable in both males and females (Table 1). Percentage body fat, TAF and SAF were higher in females as compared to males, whereas VAF area was comparable in both genders. The size of adipocytes from SC adipose tissue was greater (59.6±5.10 vs. 54.6±4.4, p<0.01) than adipocytes from OM adipose tissue, with no significant difference among the genders.

**Table 1:** Clinical, anthropometric, body composition and biochemical characteristics.

Parameter	Male (n = 40)	Female (n = 40)	P value
Age (yrs.)	39.1±10.1 (25.9)	43.6±13.1 (30.0)	0.08
Systolic blood pressure (mm Hg)	122.2±5.9 (4.8)	124.7±6.9 (5.6)	0.09
Diastolic blood pressure (mm Hg)	80.9±3.5 (4.4)	80.1±4.7 (5.9)	0.41
**Anthropometric and body composition characteristics**			
Height (cm)	169.8±5.4 (3.2)	154.1±5.0 (3.2)	<0.001
Weight (kg)	64.1±7.4 (11.6)	60.2±9.7 (16.1)	0.04
Body mass index (kg/m^2^)	22.2±2.7 (12.1)	25.6±4.5 (17.6)	<0.001
Waist circumference (cm)	80.2±6.3 (7.9)	82.3±9.2 (11.2)	0.22
Hip circumference (cm)	88.7±5.1 (5.7)	100.2±5.5 (5.6)	<0.001
Waist-to-hip ratio	0.90±.05 (5.9)	0.81±.05 (7.1)	<0.001
Biceps skinfold (mm)	7.7±2.1 (27.3)	11.0±3.2 (29.0)	<0.001
Triceps skinfold (mm)	10.5±2.8 (27.2)	19.0±4 (21.0)	<0.001
Subscapular skinfold (mm)	12.6±4.0 (32.4)	23.6±5.9 (20.9)	<0.001
Suprailiac skinfold (mm)	13.1±3.6 (27.8)	24.5±5.0 (20.5)	<0.001
Fat mass (kg)	11.4±4.7 (40.9)	20.6±5.7 (27.8)	<0.001
% Body fat	17.4±6.0 (34.4)	33.7±4.7 (14.1)	<0.001
Fat free mass (kg)	52.6±4.6 (8.9)	39.4±4.4 (11.2)	<0.001
Total abdominal fat (cm^2^)	209±68.6 (32.8)	331.3±56.5 (17.1)	<0.001
Visceral abdominal fat (cm^2^)	119.9±37.2 (31.1)	118.3±33.0 (27.9)	0.84
Subcutaneous abdominal fat (cm^2^)	72 (56, 117.5)*	197 (167, 261)*	<0.001
Subcutaneous adipocyte size (µm)	59.3±5.08 (8.5)	59.8±5.18 (8.66)	0.66
Omental adipocyte size (µm)	54.8±4.4 (8.0)	54.4±4.46 (8.2)	0.72
**Biochemical parameters**			
Fasting blood glucose (mg/dL)	82.0±8.2 (10.1)	87.8±13.9 (15.9)	0.02
Total cholesterol (mg/dL)	129.2±28.6 (22.2)	148.4±35.4 (23.9)	0.01
Serum triglycerides (mg/dL)	143.0±31.5 (22.0)	128.9±26.4 (20.5)	0.03
High density lipoprotein cholesterol (mg/dL)	39.1±5.1 (13.1)	49.9±6.3 (12.8)	<0.001
Low density lipoprotein cholesterol (mg/dL)	90.5±29.0 (33.0)	98.8±24.4 (24.7)	0.17
Fasting insulin(µU/mL)*	6.40 (3.59,16.03)	8.12 (3.83, 16.62)	0.36
HOMA-IR*	1.29 (0.68, 3.14)	1.61 (0.75, 3.80)	0.33
hsCRP (mg/L)*	1.0 (0.67, 2.3)	1.55 (0.9, 3.5)	0.11

Values in mean±SD (coefficient of variation);

* mean (interquartile range).

HOMA-IR: Homeostatic model of assessment-insulin resistance; hsCRP: high sensitivity C-reactive protein.

### Prevalence of abnormalities

Overweight was present in 42.5% males and 67.5% females (p = 0.02) whereas abdominal obesity was present in 5% males and 52.5% females (p<0.001). Prevalence of hypertension was comparable in both genders (20% and 25%, p = NS). Among the biochemical abnormalities, IFG was more prevalent in females than males (10% vs. 5%, p = 0.01). Prevalence of hypercholesterolemia (2.5% and 7.5%), hypertriglyceridemia (32.5 and 20%), low HDL-c (12.5% and 10%), high hsCRP (20% and 27.5%), high HOMA-IR (30% and 35%) and metabolic syndrome (12.5% and 27.5%) were comparable in males and females, respectively.

### Correlation of abdominal fat depots with biochemical parameters (Table 2)

**Table 2:** Spearman correlation analysis of anthropometric and biochemical parameters with abdominal fat depots.

Parameter	Total abdominal fat (Rho)		Visceral abdominal fat (Rho)		Subcutaneous abdominal fat (Rho)	
	Males (n = 40)	Females (n = 40)	Males (n = 40)	Females (n = 40)	Males (n = 40)	Females (n = 40)
Body mass index	0.56^c^	0.85^c^	0.32^a^	0.26	0.55^c^	0.81^c^
Waist circumference	0.70^c^	0.91^c^	0.59^c^	0.25	0.46^b^	0.83^c^
Waist-to-hip ratio	0.65^c^	0.84^c^	0.44^b^	0.21	0.61c	0.80^c^
% Body fat	0.54^c^	0.90^c^	0.40^a^	0.28	0.50^c^	0.81^c^
Fasting blood glucose	0.43^b^	0.50^b^	0.18	−0.01	0.44^b^	0.51^b^
Total cholesterol	0.46^b^	0.56^b^	0.21	−0.12	0.42^b^	0.62^b^
Triglycerides	0.60^c^	0.55^b^	0.38^a^	−0.05	0.62^b^	0.60^b^
High-density lipoprotein cholesterol	−0.52^b^	−0.18	−0.62^b^	−0.09	−0.33^a^	−0.09
Low-density lipoprotein cholesterol	−0.01	0.31^a^	−0.05	−0.24	0.03	0.45^b^
Fasting insulin	0.55^b^	0.59^b^	0.16	0.007	0.65^b^	0.57^b^
hsCRP	0.01	0.38^a^	0.11	0.10	−0.08	0.40^a^
HOMA-IR	0.54^b^	0.58^b^	0.16	−0.01	0.64^b^	0.57^b^

a : p<0.05;

b p<0.01;

c : p<0.001.

hsCRP: high sensitivity C-reactive protein; HOMA-IR: Homeostatic model of assessment-insulin resistance.

Total and subcutaneous abdominal fat area correlated significantly with BMI, WC, WHR and %BF in both males and females, the correlations being stronger in females. Visceral abdominal fat area correlated with BMI, WC, WHR and % BF only in males. Total abdominal fat area correlated significantly with FBG, TC, TG, fasting insulin and HOMA-IR in both genders, with hsCRP and LDL-c only in females, and correlated inversely with HDL-c only in males. Visceral abdominal fat area correlated positively with TG and inversely with HDL-c only in males. Subcutaneous abdominal fat area correlated with FBG, TC, TG, fasting insulin and HOMA-IR in both genders, with hsCRP and LDL-c only in females and inversely with HDL-c only in males.

### Correlation of adipocyte size with anthropometric and biochemical parameters

Table 3 is showing the correlation of various anthropometric and biochemical parameters with the diameter of SC and OM adipocytes. In males, BMI and WHR correlated significantly with OM adipocyte size, whereas %BF correlated significantly with both SC and OM adipocyte size. All the skinfold thicknesses correlated significantly with adipocyte size of both depots, but the correlations with OM adipocyte size were stronger. Total, subcutaneous and visceral abdominal fat area showed significant correlations with adipocyte size from both the depots. Among the biochemical parameters, only TG, insulin levels and HOMA-IR correlated significantly with OM adipocyte size.

**Table 3:** Spearman correlation analysis of anthropometric and biochemical parameters with adipocyte size.

Parameter	Subcutaneous adipocyte diameter		Omental adipocyte diameter	
	Males (n = 40)	Females (n = 40)	Males (n = 40)	Females (n = 40)
Body mass index	0.20	0.48^b^	0.35^a^	0.50^c^
Waist circumference	0.11	0.41^b^	0.22	0.61^c^
Waist-to-hip ratio	0.28	0.42^b^	0.36^a^	0.48^b^
Biceps skinfold	0.35^a^	0.18	0.44^b^	0.50^b^
Triceps skinfold	0.40^a^	−0.01	0.43^b^	0.24
Subscapular skinfold	0.36^a^	0.09	0.50^c^	0.28
Suprailiac skinfold	0.29	0.05	0.46^b^	0.15
% Body fat	0.33^a^	0.66^c^	0.32^a^	0.63^c^
Total abdominal fat	0.52^c^	0.50^c^	0.54^c^	0.56^c^
Subcutaneous abdominal fat	0.44^b^	0.49^b^	0.38^a^	0.62^c^
Visceral abdominal fat	0.43^b^	0.08	0.50^c^	−0.03
Fasting blood glucose	0.14	0.43^c^	0.26	0.44^b^
Total cholesterol	0.21	0.47^b^	0.29	0.67^c^
Serum triglyceride	0.22	0.42^b^	0.50^b^	0.52^c^
High-density lipoprotein cholesterol	−0.18	−0.17	−0.13	−0.11
Low-density lipoprotein cholesterol	−0.01	0.28	0.20	0.41^b^
Fasting insulin	0.21	0.65^c^	0.31^a^	0.62^c^
hsCRP*	−0.12	−0.17	−0.01	0.09
HOMA-IR	0.19	0.62^c^	0.29^a^	0.61^c^

a : p<0.05;

b p<0.01;

c : p<0.001.

HOMA-IR: Homeostatic model of assessment-insulin resistance; hsCRP: high sensitivity C-reactive protein.

In females, adipocyte diameters of both adipose depots showed significant correlation with BMI, WC, WHR, %BF, TAF and SAF, the correlations being stronger with OM adipocyte diameter, except for %BF. Among the skinfolds, only biceps skinfold thickness correlated with OM adipocyte diameter. Among the biochemical parameters, levels of FBG, TC, TG, insulin and HOMA-IR correlated significantly with both SC and OM adipocyte diameter (correlations of TC and TG being stronger with OM adipocyte size), whereas LDL-c levels correlated significantly only with OM adipocyte diameter. The size of adipocytes from both the abdominal fat depots was significantly higher in subjects who had metabolic syndrome as compared to those without it.

### Regression analysis

Regression analysis was performed to evaluate the influence of increase in adipocyte size of both adipose tissue depots on the biochemical parameters. In females, one unit increase in SC adipocyte size was associated with increase in 1.13 units of FBG (p = 0.007, 95% CI: 0.33–1.93), 3.34 units of TC (p = 0.001, 95% CI: 1.38–5.30), 1.90 units of TG (p = 0.01, 95% CI: 0.35–3.45), 0.13 units of fasting insulin (p<0.001, 95% CI: 0.07–0.19) and 0.14 units of HOMA-IR (p<0.001, 95% CI: 0.07–0.21). After adjusting for age, BMI, %BF, WC, TAF and SAF, only the relationship with fasting insulin (regression coefficient 0.09, p = 0.03, 95% CI: 0.008–0.17) and HOMA-IR (regression coefficient 0.10, p = 0.03, 95% CI: 0.008–0.19) remained significant whereas the relationship with FBG (regression coefficient 0.71), TC (regression coefficient 1.10) and TG (regression coefficient 0.36) became nonsignificant. In males, one unit increase in OM adipocyte size was associated with increase in 2.0 units of TC (p = 0.05, 95% CI: −0.44–4.02) and 4.0 units of TG (p<0.001, 95% CI: 2.10–5.99). This relationship was attenuated after adjusting for age, BMI, %BF, WC, TAF and SAF (TC regression coefficient 0.93, TG regression coefficient 1.73). In females, one unit increase in OM adipocyte size was associated with an increase of 1.3 units of FBG (p = 0.007, 95% CI: 0.38–2.24), 5.4 units of TC (p<0.001, 95% CI: 3.52–7.33), 2.90 units of TG (p = 0.001, 95% CI: 1.20–4.59), 0.15 units of fasting insulin (p<0.001, 95% CI: 0.07–0.22) and 0.16 units of HOMA-IR (p<0.001, 95% CI: 0.08–0.24). After adjusting for age, BMI, %BF, WC, TAF and SAF, these relationships were attenuated (FBG regression coefficient −0.26, TC regression coefficient 2.28, TG regression coefficient 0.83, fasting insulin regression coefficient 0.04 and HOMA-IR regression coefficient 0.04).

## Discussion

The observations of this study involving adult Asian Indians from India indicates that OM adipocyte size correlates more strongly with measures of generalised and abdominal obesity, abdominal fat compartments and metabolic alterations than SC adipocyte size.

A growing body of evidence suggests that variation in adipocyte size occurring in obesity has impact on adipose tissue function [29] [30]. Adipocyte hypertrophy leads to its dysfunction and reduced triglyceride storage which in turn promotes overflow of fatty acids to visceral fat and liver [7]. In addition, adiponectin secretion is reduced with increased pro-inflammatory cytokine secretion [31]. Earlier studies investigating this have included only the influence of SC adipocyte size on metabolic parameters. This may be due to the relative ease of obtaining SC adipose tissue.

There is paucity of data comparing the influence of OM and SC adipocyte size on metabolic alterations, and there are no studies performed in Asian Indians from India, to the best of our knowledge. A comparison of South Asian and Caucasian men showed that abdominal SC adipocyte size was significantly larger in South Asians and it inversely correlated with glucose disposal rate and adiponectin levels. This relationship persisted even after adjusting for SC abdominal fat mass [19]. Observations of another study comparing healthy South Asians and Caucasians revealed that South Asians had relatively higher amount of deep SC and visceral fat compared to Caucasians along with larger adipocytes [32]. Interestingly, the differences in fasting insulin, HDL-c and adiponectin levels among the two ethnic groups were not altered after adjusting for body fat distribution, but were attenuated after adjusting for adipocyte area.

In our study, a clear gender difference in the correlations of adipocyte with anthropometric and biochemical was observed. The correlations of TC, TG and LDL-c were stronger with OM than SC adipocyte size in both genders, the correlations being stronger in females. These observations are similar to an earlier study which concluded that OM adipose tissue cellularity was important predictor of hypertriglyceridemia in women. An enlargement and increase in number of OM adipocytes by 10% was associated with increased risk of hypertriglyceridemia by four times and 1.5 times, respectively. The association between OM adipocyte hypertrophy and lipid profile alterations was observed to be independent of differences in subcutaneous adipose tissue cellularity, body composition, and fat distribution in women [33].

Fasting insulin and HOMA-IR correlated significantly with TAF and SAF but not with VAF in both males and females. This is in line with earlier observations of significant association of truncal subcutaneous adipose tissue with hyperinsulinemia in Asian Indian young adults from India [34] and migrant Asian Indians [19]. However, these observations are contrary to other reports from Indian subcontinent which indicate that VAF is more closely related to insulin resistance [35]. In the present study, the correlations of SC and OM adipocyte size with fasting insulin and HOMA-IR were significantly stronger only in females. After adjusting for age, BMI, WC, %BF, TAF and SAF, only the association of fasting insulin and HOMA-IR remained significant with SC adipocyte size (p = 0.03 for both fasting insulin and HOMA-IR). These observations are similar to those reported by Weyer *et al.* in Pima Indians where mean SC abdominal adipocyte size was observed to be 19% and 11% higher in subjects with diabetes and IGT as compared to those with NGT, after adjusting for age, sex and %BF [17]. Enlarged mean SC abdominal adipocyte along with a low insulin sensitivity and acute insulin secretory response independently predicted diabetes in NGT subjects over an average follow up of about nine years. The changes in insulin sensitivity were inversely and independently related to changes in mean SC abdominal adipocyte size and %BF [17]. However, this study did not evaluate the association of OM adipocyte size with insulin sensitivity and risk of development of diabetes mellitus. In another study involving severe obese patients who underwent bariatric surgery, it was observed that OM adipocyte size correlated strongly with insulin resistance, TG/HDL-c ratio and HbA1c. The study subjects were categorised into metabolically healthy and unhealthy groups. Though mean SC adipocyte size was similar in both groups, OM adipocyte size was smaller in metabolically healthy group as compared to metabolically unhealthy group [36]. This may be explained to some extent by their greater metabolic activity and responsiveness to various hormonal alterations.

Whether adipocyte size or body composition in terms of %BF or body fat distribution predicts metabolic complications of obesity is unclear. This was investigated by Mundi *et al.* in a study involving predominantly Caucasian subjects [37]. Serum triglyceride levels were best predicted by visceral fat area in both men and women, whereas fasting insulin levels were best predicted by SC abdominal fat area in women and BMI in men. No independent association of fasting insulin with femoral adipocyte size was noted. In our study, though SC and OM adipocyte size was associated with FBG, TC, TG and insulin levels in females, this relationship was attenuated after adjusting for age, BMI, %BF, WC, TAF and SAF area of abdomen. This may further suggest that body fat distribution may have a greater influence on obesity associated metabolic abnormalities.

The strength of our study consists is that we have evaluated the relationship of adipocyte size from abdominal SC and OM fat depots with measures of generalised and abdominal obesity, abdominal fat distribution, and various biochemical parameters in Asian Indians from India. Some of our observations are similar to those reported earlier such as strong relationship of SC adipocyte size with insulin resistance and that of OM adipocyte size with dyslipidemias, with clear gender differences. This study has some limitations. The relatively small sample size may not be sufficient to draw firm conclusions. Moreover, the prevalence of abdominal obesity was very low in males in this study. This could be related to the sample selection as the subjects were included from individuals undergoing elective surgery and may not be representative of general population. However, this study is first of its kind involving Asian Indians from India and may be helpful in planning further studies involving larger number of subjects. Due to logistic reasons, oral glucose tolerance test could not be performed in the study. We used HOMA-IR as a surrogate marker of insulin resistance instead of clamp study due to limitations of infrastructure. However, HOMA-IR has been used as a valid surrogate marker of insulin resistance by several investigators. Further studies involving larger number of subjects with wide range of BMI, more accurate measures and higher number of parameters are required to draw firm conclusions.

In conclusion, in this study involving Asian Indians from India, the OM adipocyte size, though smaller than SC adipocyte, was more closely related to measures of generalised as well as abdominal obesity and metabolic abnormalities with distinct gender differences. The relationship of adipocyte size with metabolic parameters was attenuated after adjusting for measures of adiposity.

## Supporting Information

Data S1 **Raw data of the study population.** The raw data of the anthropometric, biochemical and adipocyte size of the study population.
(XLSX)
